# Metabolic Signatures of Aging and Gametogenesis in *Hydra oligactis*

**DOI:** 10.1111/acel.70643

**Published:** 2026-07-27

**Authors:** Nicki Marami‐Zonouz, Erwann Arc, Thomas Roach, Pidder Jansen‐Dürr, Werner Bader, Bert Hobmayer, Ilse Kranner

**Affiliations:** ^1^ Department of Botany University of Innsbruck Innsbruck Austria; ^2^ Institute for Biomedical Aging Research University of Innsbruck Innsbruck Austria; ^3^ Institute of Zoology University of Innsbruck Innsbruck Austria

**Keywords:** aging, Cnidaria, hydra, metabolite profiling, metabolomics, senescence, sexual reproduction, taurine

## Abstract

Aging can be experimentally induced in *Hydra oligactis* , making it an exception among the “immortal” cnidarian genus *Hydra*. In response to cold temperatures, *H. oligactis* polyps switch from asexual to “emergency” sexual reproduction and eventually age and die. We used GC–MS‐based metabolite profiling to characterize cold‐induced (CI) metabolic reprogramming during concurrent sexual differentiation and aging in *H. oligactis* . Metabolites in four clusters either decreased or increased progressively until week 8, when animals were severely aged, whereas others peaked at sexual maturity after 4–6 weeks. At week 4, signatures of failing cytoprotection and neurotransmitter function appeared in both sexes, including a drastic reduction of taurine, whose deficiency is a known driver of aging in other organisms. Taurine supplementation partly reversed interstitial stem cell loss during the first 2 weeks of cold induction and subsequent sexual differentiation. Metabolites in the NAD^+^ synthesis and salvage pathways, such as picolinate and niacin, also declined. Changes in pyruvate and TCA cycle intermediates indicated increased energy demands associated with both gametogenesis and aging. Urate, the final oxidation product of purine metabolism, showed the highest fold‐change, peaking at sexual maturity in both sexes and remaining elevated. The abundance of most purine and pyrimidine bases also increased, in line with nucleotide cleavage by apoptosis, as part of gametogenesis and aging. Sexual maturity was clearly reflected by high levels of polyamines, such as putrescine, cadaverine, and 3‐hydroxybutyrate, required for spermatogenesis. Our study reveals metabolic processes related to gametogenesis and aging and highlights differences between female and male metabolism.

## Graphical abstract

*Hydra oligactis* undergoes cold‐induced sexual differentiation and aging, culminating in death. GC–MS‐based metabolite profiling revealed signatures of gametogenesis, for example, polyamine accumulation, and of aging, such as loss of cytoprotection and neurotransmitter function, including taurine depletion. Taurine supplementation partly rescued stem‐cell loss and induced a shift from sexual to asexual reproduction.

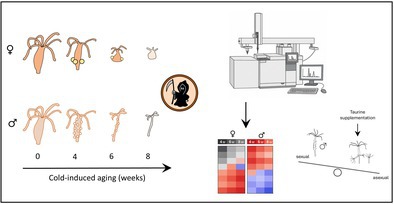

**Abbreviations**
CI: cold‐induced
GABA: γ‐aminobutyric acid
GC–MS: gas chromatography coupled to mass spectrometry
PCA: principal component analysis

Aging is a multifaceted process characterized by physiological decline, culminating in increased vulnerability and death (López‐Otín et al. 2023). While accumulation of molecular damage partly explains this functional decline (Harman 1956), aging is developmentally programmed in many species (de Magalhães 2012; Gems 2022). Hallmarks of aging include genomic instability, telomere attrition, macromolecular damage, impaired nutrient sensing, mitochondrial dysfunction and stem cell exhaustion (López‐Otín et al. 2023). Aging phenomena vary between species and kingdoms (Ackermann et al. 2007) and even non‐aging species, including *Hydra*, appeared across the tree of life (Jones et al. 2014). *Hydra*, a model for regeneration and pattern formation research (Galliot 2012), consists of three adult stem cell lineages with high self‐renewal capacity. All stem cells continuously proliferate and can differentiate into specialized cell types migrating to the body ends and newly emerging buds (Bosch et al. 2010), allowing asexual reproduction without senescence or aging over years of laboratory culture under constant conditions (Martínez 1998; Schaible et al. 2015).

However, in *H. oligactis* , low temperatures trigger a shift from asexual budding to gametogenesis, after which the parent polyps age, degenerate, and die (Brien 1953), which has been correlated with stress‐response capacity, including impaired induction of molecular chaperones such as HSP70 proteins (Bosch et al. 1988; Brennecke et al. 1998). Aging and gametogenesis are closely associated in *H. oligactis* , as cold‐induced sexual differentiation coincides with progressive parental degeneration in nearly all laboratory strains analyzed to date (Schenkelaars et al. 2018). A minor fraction of polyps does not respond to cold induction with sexual differentiation, shows no signs of degeneration, and remains asexual. Similar to many other species, *Hydra* aging involves autophagy deficiency, impaired damage repair, increased stem cell death, nervous system disorganization, musculature degeneration, and reduced regeneration and feeding abilities (Sebestyén et al. 2018; Sun et al. 2020; Tomczyk et al. 2019, 2020; Yoshida et al. 2006). With these aging phenotypes, *H. oligactis* is an emerging ancestral aging model.

Using clonal polyps mass‐cultured at 18°C as controls, we investigated cold (10°C)‐induced (CI) changes in body size and metabolism using GC–MS‐based metabolite profiling (details in Supporting Information). Cold induction reproducibly elicits an aging phenotype in *H. oligactis* , whereas hydroxyurea—previously suggested as an alternative trigger to induce aging (Tomczyk et al. 2020)—caused rapid interstitial stem‐cell depletion and mortality, reflecting acute cytotoxicity rather than gradual aging (Figure 1a; Figure S1). Cold induction, by contrast, induced progressive viability loss over 16 weeks (Figure 1a) and was therefore adopted as a physiologically relevant aging paradigm for all subsequent experiments. Cell loss occurred early upon cold induction, as described before (Yoshida et al. 2006). Within 6 weeks, epithelial cell numbers and body and tentacle sizes decreased in both sexes. By week 8, epithelial cell numbers dropped to 46% and 32% in females and males, respectively, while body size shrank to 18% and 78%, respectively, suggesting that male bodies remained elongated despite low cell numbers (Figure 1a). By week 4, eggs and testes appeared on the body columns. Females were fully sexually mature at week 6 and males at week 4 (Figure 1b). Polyps aged for 0, 4, 6, and 8 weeks displayed distinct metabolite profiles (Figures 1c and 2a). Differentially accumulated metabolites were separated into four clusters, progressively decreasing or increasing, or peaking at sexual maturity (Figure 2b).

**FIGURE 1:**
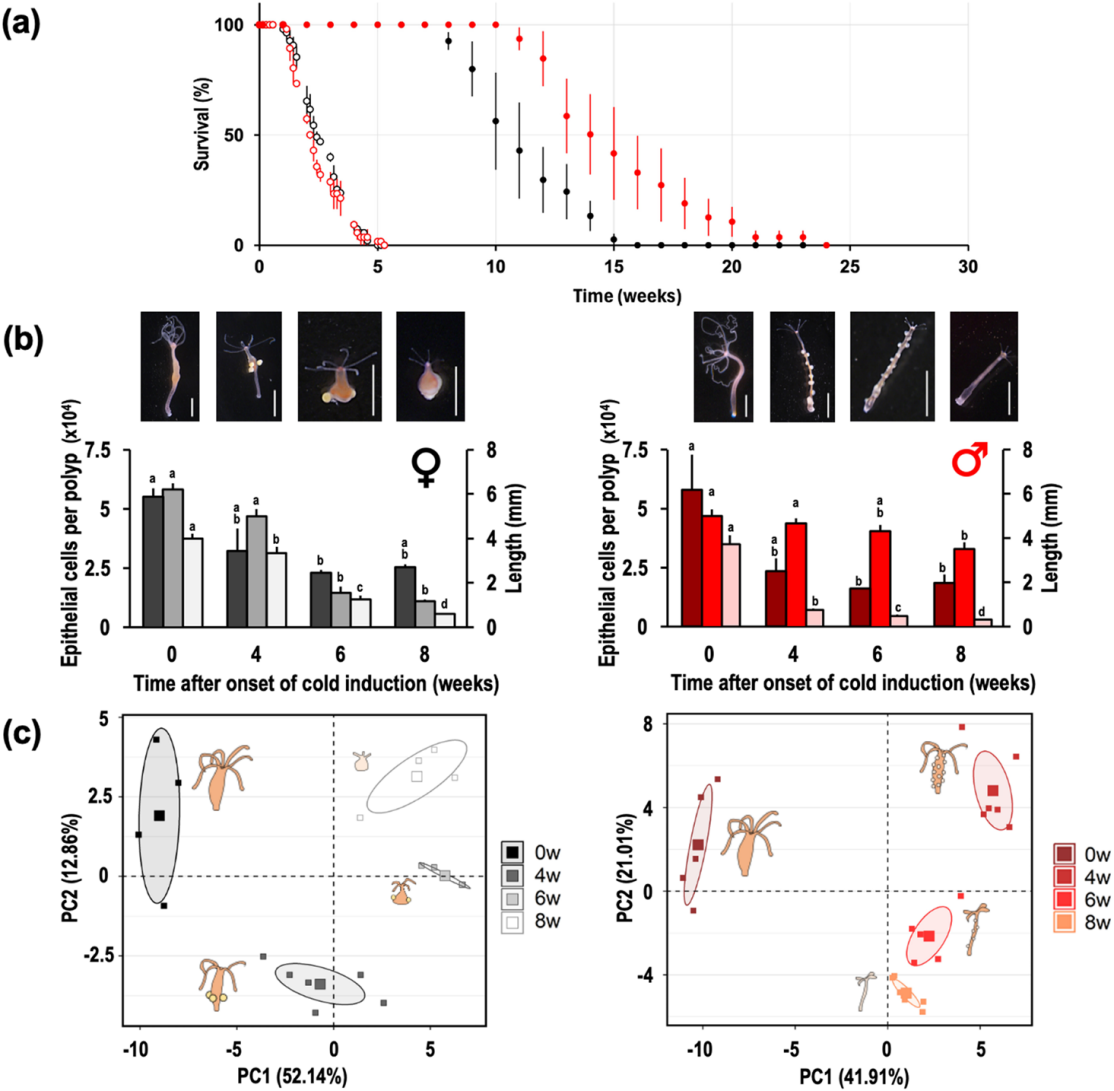
Effects of cold induction on *H. oligactis* morphology and physiology. Gray and red shades denote female and male polyps, respectively. (a) Kaplan–Meier survival curves of female (black) and male (red) *H. oligactis* after cold induction (closed circles) compared to hydroxyurea treatment (open circles) at 18°C. (b) Epithelial cell number (*N* = 3 × 10 polyps, dark shades), body and tentacle length (medium and light shades; data are means + SE; *N* = 11–13 polyps); different letters indicate significant differences; *p* < 0.05; scale bars: 2 mm. (c) PCA analysis of differentially accumulated, log‐transformed metabolites. Time intervals are shown by dark‐to‐light shades; polyp schematics depict developmental stages with shrinking body size between 0 and 8 weeks of cold induction. Larger symbols within ellipses denote the centroids (means) and smaller symbols represent the individual replicates (*N* = 4–6 replicates).

**FIGURE 2:**
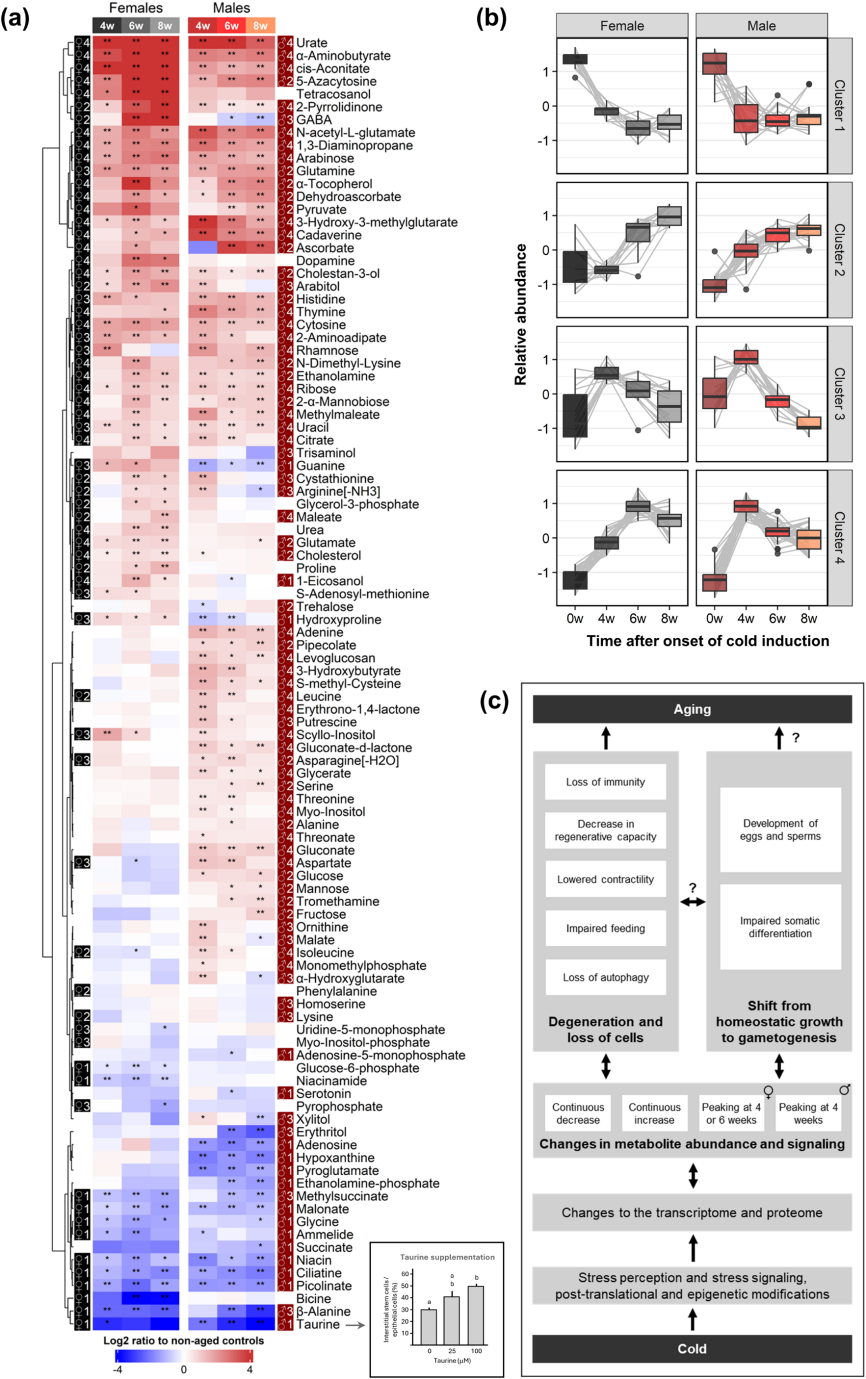
Effects of cold induction on *H. oligactis* metabolism. (a) Fold changes of differentially accumulated metabolites, relative to week 0, on a log_2_ color scale (bottom). Values are means of 4–6 biological replicates. One and two asterisks denote FDR‐adjusted *p*‐values of < 0.05 and 0.01, respectively. The bottom inset shows the increase in the interstitial stem‐cell to epithelial‐cell ratio after taurine supplementation of male polyps aged for 2 weeks under cold induction plus continuous taurine treatment (*N* = 3 × 5 macerated large budless polyps; different letters indicate significant differences; *p* < 0.05). (b) Clustering of metabolites (details in Tables S1 and S2). (c) Simplified model of the effects of cold induction. We propose that stress perception affects gene expression, resulting in metabolic changes, which along with post‐translational and epigenetic modifications alter signaling processes leading to aging and gametogenesis.

Taurine, a widespread non‐proteinogenic amino sulfonic acid whose deficiency has been linked to metabolic impairment, including mitochondrial dysfunction, tissue degeneration, and shortened lifespan (Singh et al. 2023), dropped in both sexes by week 4 (Figure 2a). Taurine suppresses senescence and DNA damage and affects DNA methylation in vertebrates, worms, and yeast. In mammals, taurine and its derivatives maintain healthy stem cells, and supplementation can improve life‐ and healthspan (Barua et al. 2001; Jacobsen and Smith 1968; Singh et al. 2023). In the cnidarian *Cyanea capillata* , taurine may act as a neurotransmitter (Anderson and Trapido‐Rosenthal 2009). Taurine and β‐alanine (depleted by week 4 in females; week 6 in males) are involved in prolonged mouth opening in *Hydra* (Pierobon et al. 2001). Notably, in CI polyps, taurine supplementation restored the interstitial stem‐cell to epithelial‐cell ratio and shifted the balance from sexual toward asexual reproduction, supporting an anti‐aging effect of taurine (Figure 2a; Figure S2). Aging is also characterized by increased stem‐cell degeneration in mice, which can be counteracted by activating the NAD^+^ salvage pathway through supplementation with nicotinamide riboside, a precursor of NAD^+^ (Zhang et al. 2016) or by taurine supplementation (Singh et al. 2023). Glycine, a cytoprotectant (Weinberg et al. 1987), also dropped in both sexes of cold‐induced *H. oligactis* . Glycine supplementation improves lifespan in mice and worms (Liu et al. 2019; Miller et al. 2019), and glycine N‐methyltransferase modulates lifespan in flies (Obata and Miura 2015). In CI *H. oligactis* , loss of neurogenesis includes lowered responses of hypostomal neural circuits to glycine and γ‐aminobutyric acid (GABA) and glutamate (Pierobon et al. 2004; Tomczyk et al. 2019), which increased in both sexes, although bacteria accumulating around aging *Hydra* may also produce GABA (Pierobon 2021). In summary, metabolic signatures of aging and neurotransmitter imbalance emerged already at week 4.

NAD^+^ depletion is another hallmark of aging observed in animal models (Fang et al. 2017), and NAD^+^ precursors improve life‐ and healthspan in yeast, *C. elegans* and mice (Belenky et al. 2007; Fang et al. 2016; Zhang et al. 2016). At week 4, niacin (or nicotinic acid), a precursor of NAD^+^ and NADP^+^ in the Preiss‐Handler pathway (Romani et al. 2019) and picolinate, a by‐product of NAD^+^ production from tryptophan, dropped in both sexes (Figure 2a). In the human central nervous system, altered picolinate production is linked to age and disease (Coggan et al. 2009; Tan et al. 2012). Niacin and niacinamide (or nicotinamide) feed into the NAD^+^ salvage pathway, supporting NAD^+^ biosynthesis in aged mice (Katsyuba and Auwerx 2017). The early drop in picolinate and niacin in both sexes, and niacinamide in females, further underlines that aging had started by week 4.

Changes to the tricarboxylic acid (TCA) cycle also contribute to aging. For example, citrate can form from glutamine in defective mitochondria via reverse TCA cycle activity in aging or tumor cells (Mullen et al. 2011; Sun et al. 2016). Glutamine and glutamate levels also increased in CI *H. oligactis* . Likewise, glutamate accumulation via enhanced glutaminolysis protects senescent human cells and mouse tissues from cell death (Johmura et al. 2021). Other TCA cycle intermediates may also have entered the TCA cycle via anaplerotic reactions. For example, maleate can be hydroxylated to malate. Increased energy demands were reflected by rising or peaking levels of pyruvate, citrate, and cis‐aconitate, which supports mouse oocyte maturation by affecting redox homeostasis (Li et al. 2020), while succinate declined in both sexes (Figure 2a).

As aging and gametogenesis both require energy, interpreting variations in TCA cycle activity remains intricate. However, signatures of sexual reproduction were evident in polyamines. Cadaverine, putrescine, spermine, and spermidine influence cell proliferation, growth, oogenesis, spermatogenesis, and embryogenesis in mammals (Coffino 2000; Lefevre et al. 2011; Wallace et al. 2003). Putrescine levels rise in mammalian Sertoli cells, which provide energy for germ cell motility via ketones like 3‐hydroxybutyrate (Jutte et al. 1985; Tanaka et al. 2004), produced when excess acetyl‐CoA forms from fatty acid oxidation (Casals et al. 1992; Hegardt 1999). In *Hydra*, epithelial cells may have similar roles as Sertoli cells in sperm precursor development (Kuznetsov et al. 2001). Thus, peak levels of putrescine, cadaverine, and 3‐hydroxybutyrate at male, but not female, sexual maturity reflected spermatogenesis in the fully differentiated germ cells (Figure 1a), supported by lysine and arginine and ornithine (peaking at week 4), from which cadaverine and putrescine (Figure 2a), respectively, are produced by decarboxylation.

Gametogenesis also requires apoptosis. In male *Hydra*, apoptotic cells appear in testes during spermatogenesis and are phagocytized by epithelial cells (Kuznetsov et al. 2001). In female *Hydra*, germ cells differentiate into one oocyte and thousands of nurse cells undergoing unusual apoptosis. After phagocytosis by the growing oocyte, apoptosis is arrested until the new polyp hatches (Technau et al. 2003). Apoptosis involves nucleic acid cleavage, and purines can be degraded to urate, the metabolite with the highest fold‐change at sexual maturity in both sexes. High urate levels are also consistent with urate acting as a danger signal (Shi et al. 2003). Hypoxanthine, dropping at week 4 in males (Figure 2a), can be oxidized into xanthine and further converted to urate (Glantzounis et al. 2005). Xanthine oxidase may induce aging‐related oxidative stress (Ryan et al. 2011), and hypoxanthine supplementation increases nematode lifespan (Copes et al. 2015). The purine bases, guanine and adenine, peaked in females and males, respectively, while guanine and adenosine levels dropped at week 4 in males. The pyrimidine bases, uracil, thymine and cytosine, peaked or increased in both sexes (Figure 2a). Old nematodes also have low adenine, adenosine and guanine levels. Overall, the CI variations in purine and pyrimidine bases bear signatures of gametogenesis and aging.

In conclusion, we (1) show that CI biological processes in *H. oligactis* (Figure 2c) are reflected by distinct metabolic fingerprints, a step‐change toward relating aging in *Hydra* to aging in other animals, and (2) revealed common denominators—including an association of taurine with longevity‐related traits—but also sex‐specific differences, which are important as female metabolism still receives less attention than male metabolism by mainstream biomedical sciences. (3) *Hydra* shares a broad genetic toolkit with vertebrates, including humans, and represents a simple metazoan model for whole‐organism aging. Therefore, studying *H. oligactis* mutants may aid future research on genetic regulation of gametogenesis and aging.

## Author Contributions

P.J.‐D., I.K. and B.H. conceived the project and acquired the funding; N.M.‐Z., B.H. and I.K. designed the study, with contributions from T.R. and E.A.; N.M.‐Z. carried out experiments, supported by W.B., and analyzed the data, supported by E.A.; N.M.‐Z. and I.K. wrote the first draft of the manuscript; all authors contributed to writing the manuscript.

## Funding

This work was funded by the European Commission (COFUND‐ARDRE, Project 847681; DOI: https://doi.org/10.3030/847681), and by the Doctoral Program Ageing and Regeneration (DP AGE_REG) of the University of Innsbruck. Open access funding provided by Universitat Innsbruck/KEMÖ.

## Conflicts of Interest

The authors declare no conflicts of interest.

## Supporting information


**Figure S1:** Comparison of the effects of cold induction and hydroxyurea treatment on the interstitial stem‐cell to epithelial‐cell ratio in *Hydra oligactis* polyps. Hydroxyurea (open symbols) caused an acute loss of interstitial stem cells, irrespective of sex, whereas the ratio declined only gradually during cold induction (closed symbols), slightly faster in female (black) than male (red) polyps.
**Figure S2:** Effects of taurine on sexual and asexual reproduction in male *Hydra oligactis* polyps. Supplementation with 100 μM taurine during the first 3 weeks of cold induction reduced (a) the proportion of sexually differentiated male versus non‐responding polyps, and (b) the number of testes per male polyp. In contrast, (c) the average number of buds per polyp increased in taurine‐treated cohorts. (d) Bud developmental stage was not significantly affected, indicating that taurine promoted bud induction rather than growth. Bars in (a) to (d) represent mean ± SD from three independent experiments (*n* = 30–38 large budless polyps from the male strain placed in medium‐sized Petri dishes); different letters indicate significant differences (*p* < 0.05). (e) Schematic illustration of the taurine‐induced shift from sexual toward asexual reproduction, consistent with an anti‐aging effect of taurine.

**Table S1:** Full data set of metabolites found by GC–MS‐based metabolite profiling, including statistical evaluation.

**Table S2:** Metabolites with significant changes in abundance in CI female and male *H. oligactis* . Coseq clustering revealed 4 clusters: the abundance of metabolites in clusters 1 and 2 increased and decreased, respectively, between week 0 and week 8, and those in cluster 3 and 4 lists peaked at 4 or 6 weeks.

## Data Availability

The metabolite data that supports the findings of this study are available in the Supporting Information of this article. Animal physiology data are available from the corresponding author upon request.
