# Supplementary material for: Metabolic Signatures of Aging and Gametogenesis in Hydra oligactis
Source: Aging Cell. 2026 Jul 27;25(8):e70643. doi: 10.1111/acel.70643 (PMC13403047; doi:10.1111/acel.70643)
Supplement: Supplementary file 1 — Figure S1: Comparison of the effects of cold induction and hydroxyurea treatment on the interstitial stem‐cell to epithelial‐cell ratio in Hydra oligactis polyps. Hydroxyurea (open symbols) caused an acute loss of interstitial stem cells, irrespective of sex, whereas the ratio declined only gradually during cold induction (closed symbols), slightly faster in female (black) than male (red) polyps. Figure S2: Effects of taurine on sexual and asexual reproduction in male Hydra oligactis polyps. Supplementation with 100 μM taurine during the first 3 weeks of cold induction reduced (a) the proportion of sexually differentiated male versus non‐responding polyps, and (b) the number of testes per male polyp. In contrast, (c) the average number of buds per polyp increased in taurine‐treated cohorts. (d) Bud developmental stage was not significantly affected, indicating that taurine promoted bud induction rather than growth. Bars in (a) to (d) represent mean ± SD from three independent experiments (n = 30–38 large budless polyps from the male strain placed in medium‐sized Petri dishes); different letters indicate significant differences (p < 0.05). (e) Schematic illustration of the taurine‐induced shift from sexual toward asexual reproduction, consistent with an anti‐aging effect of taurine. [file ACEL-25-e70643-s001.docx]

**Supporting Information**

**Methods**

**Culturing, aging, quantification of epithelial cells and body size, and sampling**

Two strains of *H. oligactis*, Innsbruck female12 (Cazet et al., 2023) and Innsbruck male1, originally collected from Lake Piburg, Tyrol, Austria (47° 11′ 42″ N, 10° 53′ 20″ O; 913 m a.s.l.), and raised clonally from a single individual each, were used. Polyps were maintained in asexual mass culture in *Hydra* medium (1.0 mM CaCl_2_, 1 mM NaHCO_3_, 0.1 mM MgCl_2_, 0.03 mM KNO_3_, 1 mM Tris HCl pH 7.8) at 18 °C and fed five times a week (Hobmayer, Holstein, & David, 1997).

In *H. oligactis*, cold induction reproducibly elicits an aging phenotype (Brien, 1953). Hydroxyurea treatment has been suggested as an alternative means to induce aging (Tomczyk et al., 2020), as it ablates fast-cycling interstitial stem cells (Sacks & Davis, 1979), producing epithelial polyps composed solely of slow-cycling epithelial cells and thereby preventing gametogenesis. In our analyses, however, hydroxyurea caused rapid stem-cell depletion within one week (Fig. S1) and mortality shortly thereafter (Fig. 1a), with death of the first individual in the cohort observed at day 7 and 8, in females and males, respectively, and of the last at day 35 and 37, respectively, consistent with acute cytotoxicity rather than gradual aging. Cold induction, by contrast, triggered progressive senescence over 16 weeks, following a classical Kaplan–Meier pattern, with death of the first individual in the cohort observed at day 56 and 77 days in females and males, respectively, and of the last at day 112 and 168 (Fig. 1a). Upon cold induction, interstitial stem-cell numbers declined by two-thirds within six weeks and then slowly to about one-sixth of the initial level after 16 weeks (Fig. S1). We therefore consider cold induction the more physiologically relevant paradigm of aging in *H. oligactis* and used it for all subsequent experiments. For cold induction, adult budless polyps were transferred to 10 °C, and fed once a week. After 4, 6 and 8 weeks, cold-induced (CI) animals were collected ≥24 h after feeding, flash-frozen in liquid nitrogen and freeze-dried. Animals maintained at 18°C were collected as controls (week 0). After 4 weeks, clear cohorts of male and female polyps could be identified, avoiding contamination by non-responders. At 4 and 6 weeks, peak sexual maturity occurred in males and females, respectively, whereas at 8 weeks advanced aging was evident before the death of the first individuals, avoiding the mixing of aging and dying polyps.

For body and tentacle length measurements, 12 budless adult polyps were anesthetised in 2% linalool for 5 min at each aging interval. Animals were photographed using a Leica MZ16F dissecting microscope and images analyzed using ImageJ (version 1.53s). For the quantification of epithelial cell number, maceration preparation of aging polyps was conducted as previously described (Hobmayer et al., 1997), using 10 polyps for each biological replicate. For performing cell counts, maceration was performed according to David (1973).

**Hydroxyurea and taurine treatments**

Hydroxyurea treatment was conducted as described by (Sacks & Davis, 1979) using 10 mM hydroxyurea during cold induction.

To test the effects of taurine on interstitial stem cells, male polyps were aged for two weeks under cold induction and supplementing the Hydra medium with 0, 25 or 100 μM taurine for two weeks. To assess the effects of taurine on sexual versus asexual reproduction, male polyps were maintained under cold induction with 0 or 100 µM taurine for three weeks, allowing male individuals to be clearly identified. To ensure continuous exposure, and given the known stability of taurine in aqueous solution, taurine-containing medium was replaced with freshly prepared solution five times per week.

**Metabolite profiling**

GC-MS-based metabolite profiling was performed using the method described by Fiehn (2016) with minor modifications. Six biological replicates were analyzed, except for females aged for 6 weeks and 8 weeks (4 and 5 replicates, respectively) and for males aged for 6 weeks (5 replicates), for which insufficient material for 6 replicates was available. Briefly, freeze-dried samples were weighed into tubes (5.0 ± 0.1 mg for males and 3.0 ± 0.1 mg for females, corresponding to approximately 50-100 polyps for females and 150-160 polyps for males per biological replicate) containing two 5 mm glass beads, and ground on pre-cooled racks using a TissueLyser (Qiagen, Hilden, Germany) at 30 Hz for 3 min.

Metabolites were extracted by adding 500 µl for males and 300 µl for females of water:acetonitrile:isopropanol (2:3:3) containing 21.25 µM isotopically labelled sorbitol (^13^C_6_) and 25 μM valine (^13^C_5_,^15^N) as internal standards. Tubes were shaken at 1,200 rpm at 4 °C for 5 min before being transferred to the Tissue-Lyser and homogenized at 22 Hz for 2 min while the samples were kept cold. Solid material was removed by centrifugation at 20,000 g for 5 min, and 25 μl of supernatant was collected and evaporated in a vacuum centrifuge (SpeedVac SPD111, Thermo Fisher Scientific Inc., Waltham, MA, USA) at room temperature for 3 h. Metabolites were derivatized using 10 μl of 20 g l^−1^ methoxyamine hydrochloride in pyridine and shaken at 600 rpm on a thermomixer (Eppendorf, Hamburg, Germany) at 28 °C for 90 min. Then, 90 μl of N-methyl-N-trimethylsilyl-trifluoroacetamide (MSTFA) was added and samples were incubated and shaken on a thermomixer at 600 rpm at 37 °C for 30 min. Samples were transferred to vials, flushed with nitrogen and allowed to equilibrate to room temperature prior to analysis.

Starting 2 hours after derivatisation, 1 µl of each sample was injected in the split-splitless inlet of a 7890B gas chromatograph (Agilent Technologies, Santa Clara, CA, USA), operated at 250 °C in splitless mode. Analytes were separated on a 30 m Rxi-5Sil MS column with a 10 m Integra-Guard pre-column (Restek, Bellefonte, PA, USA) using helium as carrier gas at 1 ml min^−1^. The GC oven temperature was initially set to 70 °C for 7 min, then ramped up by 10 °C min^−1^ to 325 °C, which was held for 10 min. Mass spectra were acquired using a Pegasus BT time of flight mass spectrometer (LECO Corporation, St. Joseph, MI, USA), scanning from 50 to 550 m/z at a frequency of 15 spectra s^−1^. The transfer line and ion source temperatures were set to 290 and 250 °C, respectively. Between consecutive injections, the 10 μl syringe was washed four times each with hexane and ethyl acetate. A mix of alkanes dissolved in 2 mg l^-1^ of hexane was injected in the middle of the queue to allow for the determination of Kováts' alkane-based retention indices (Kovats, 1958). Data acquisition and review was conducted using ChromaTof (version 5.56.57, LECO) in combination with the National Institute of Standards and Technology (NIST, 2020 release), Golm and Fiehn mass spectral libraries for compound identification (Kind et al., 2009; Kopka et al., 2005). Identifications were individually checked and assigned a confidence score based on the strength of the match of both spectral data and retention indices. Relative metabolite abundances were calculated by normalising the peak areas for compound-specific fragments to these of the labelled internal standards and to the dry weight of sample material used. Replicate samples of female and male polyps were processed separately.

**Statistical analyses**

Significance of changes in body size and epithelial cell number (Fig. 1), effects of taurine on interstitial stem cells (Fig. 2) and sexual *versus* asexual reproduction (Fig. S2) was tested by the Kruskal-Wallis test after adjustment by Bonferroni correction with significance level p<0.05. Statistical analysis of metabolite profiling data was conducted with R (R Core Team, 2024), using the RFLOMICS package developed by INRAE available in Github (https://github.com/RFLOMICS/RFLOMICS). Briefly, differentially accumulated metabolites were selected using the LIMMA package (Ritchie et al., 2015), after median scaling and log2 transformation. P-values obtained for the different time intervals were adjusted for multiple testing using false discovery rate (FDR) correction (Benjamini & Hochberg, 1995). The heatmap was generated for differentially accumulated metabolites using the ComplexHeatmap package (Gu, 2022; Gu, Eils, & Schlesner, 2016), using the Euclidean distance and Ward’s clustering method for row hierarchical clustering. Metabolites clusters were generated using the coseq package (Godichon-Baggioni, Maugis-Rabusseau, & Rau, 2019; Rau & Maugis-Rabusseau, 2018).

**Use of Artificial Intelligence Tools**

ChatGPT was used to generate schematic illustrations of GC–MS, heatmap and grim reaper in the graphical abstract, and to assist in stylistic refinement by shortening selected complex sentences.

**Supporting Figures**

Figure S1. Comparison of the effects of cold induction and hydroxyurea treatment on the interstitial stem-cell to epithelial-cell ratio in *Hydra oligactis* polyps. Hydroxyurea (open symbols) caused an acute loss of interstitial stem cells, irrespective of sex, whereas the ratio declined only gradually during cold induction (closed symbols), slightly faster in female (black) than male (red) polyps.

Figure S2. Effects of taurine on sexual and asexual reproduction in male *Hydra oligactis* polyps. Supplementation with 100 μM taurine during the first three weeks of cold induction reduced (a) the proportion of sexually differentiated male *versus* non-responding polyps, and (b) the number of testes per male polyp. In contrast, (c) the average number of buds per polyp increased in taurine-treated cohorts. (d) Bud developmental stage was not significantly affected, indicating that taurine promoted bud induction rather than growth. Bars in (a) to (d) represent mean ± SD from three independent experiments (n= 30-38 large budless polyps from the male strain placed in medium-sized Petri dishes); different letters indicate significant differences (p < 0.05). (e) Schematic illustration of the taurine-induced shift from sexual toward asexual reproduction, consistent with an anti-aging effect of taurine.

**Supporting Tables**

Table S1. Full data set of metabolites found by GC-MS-based metabolite profiling, including statistical evaluation.

Table S2. Metabolites with significant changes in abundance in cold-induced female and male *H. oligactis*. Coseq clustering revealed 4 clusters: the abundance of metabolites in clusters 1 and 2 increased and decreased, respectively, between week 0 and week 8, and those in cluster 3 and 4 lists peaked at 4 or 6 weeks.

**Supporting References**

Benjamini, Y., & Hochberg, Y. (1995). Controlling the false discovery rate: A practical and powerful approach to multiple testing. *Journal of the Royal Statistical Society: Series B (Methodological), 57*(1), 289-300. doi:<https://doi.org/10.1111/j.2517-6161.1995.tb02031.x>

Brien, P. (1953). La Pérennité Somatique. *Biological Reviews, 28*, 308-349. doi:<https://doi.org/10.1111/j.1469-185X.1953.tb01381.x>

Cazet, J. F., Siebert, S., Little, H. M., Bertemes, P., Primack, A. S., Ladurner, P., . . . Juliano, C. E. (2023). A chromosome-scale epigenetic map of the *Hydra* genome reveals conserved regulators of cell state. *Genome Research, 33*(2), 283-298. doi:<https://doi.org/10.1101/gr.277040.122>

David, C. N. (1973). A quantitative method for maceration of *Hydra* tissue. *Wilhelm Roux' Archiv für Entwicklungsmechanik der Organismen, 171*(4), 259-268. doi:<https://doi.org/10.1007/BF00577724>

Fiehn, O. (2016). Metabolomics by gas chromatography–mass spectrometry: combined targeted and untargeted profiling. *Current Protocols in Molecular Biology, 114*(1), 30.34.31-30.34.32. doi:<https://doi.org/10.1002/0471142727.mb3004s114>

Godichon-Baggioni, A., Maugis-Rabusseau, C., & Rau, A. (2019). Clustering transformed compositional data using K-means, with applications in gene expression and bicycle sharing system data. *Journal of Applied Statistics, 46*(1), 47-65. doi:<https://doi.org/10.1080/02664763.2018.1454894>

Gu, Z. (2022). Complex heatmap visualization. *Imeta, 1*(3), e43. doi:<https://doi.org/10.1002/imt2.43>

Gu, Z., Eils, R., & Schlesner, M. (2016). Complex heatmaps reveal patterns and correlations in multidimensional genomic data. *Bioinformatics, 32*(18), 2847-2849. doi:<https://doi.org/10.1093/bioinformatics/btw313>

Hobmayer, B., Holstein, T. W., & David, C. N. (1997). Stimulation of tentacle and bud formation by the neuropeptide head activator in *Hydra magnipapillata*. *Developmental Biology, 183*(1), 1-8. doi:<https://doi.org/10.1006/dbio.1996.8491>

Kind, T., Wohlgemuth, G., Lee, D. Y., Lu, Y., Palazoglu, M., Shahbaz, S., & Fiehn, O. (2009). FiehnLib: mass spectral and retention index libraries for metabolomics based on quadrupole and time-of-flight gas chromatography/mass spectrometry. *Analytical Chemistry, 81*(24), 10038-10048. doi:<https://doi.org/10.1021/ac9019522>

Kopka, J., Schauer, N., Krueger, S., Birkemeyer, C., Usadel, B., Bergmüller, E., . . . Stitt, M. (2005). GMD@ CSB. DB: the Golm metabolome database. *Bioinformatics, 21*(8), 1635-1638. doi:<https://doi.org/10.1093/bioinformatics/bti236>

Kovats, E. (1958). Gas‐chromatographische charakterisierung organischer verbindungen. Teil 1: retentionsindices aliphatischer halogenide, alkohole, aldehyde und ketone. *Helvetica Chimica Acta, 41*(7), 1915-1932. doi:<https://doi.org/10.1002/hlca.19580410703>

Rau, A., & Maugis-Rabusseau, C. (2018). Transformation and model choice for RNA-seq co-expression analysis. *Briefings in Bioinformatics, 19*(3), 425-436. doi:<https://doi.org/10.1093/bib/bbw128>

Ritchie, M. E., Phipson, B., Wu, D., Hu, Y., Law, C. W., Shi, W., & Smyth, G. K. (2015). LIMMA powers differential expression analyses for RNA-sequencing and microarray studies. *Nucleic Acids Research, 43*(7), e47-e47. doi:<https://doi.org/10.1093/nar/gkv007>

Sacks, P. G., & Davis, L. E. (1979). Production of nerveless *Hydra* *attenuata* by hydroxyurea treatments. *Journal of Cell Science, 37*(1), 189-203. doi:<https://doi.org/10.1242/jcs.37.1.189>

Tomczyk, S., Suknovic, N., Schenkelaars, Q., Wenger, Y., Ekundayo, K., Buzgariu, W., . . . Galliot, B. (2020). Deficient autophagy in epithelial stem cells drives aging in the freshwater cnidarian *Hydra*. *Development, 147*(2). doi:<https://doi.org/10.1242/dev.177840>
